# Efficacy and safety of hydromorphone for cancer pain: a systematic review and meta-analysis

**DOI:** 10.1186/s12871-024-02638-y

**Published:** 2024-08-09

**Authors:** Mohammadreza Alinejadfard, Shahryar Rajai Firouzabadi, Ida Mohammadi, Soroush Oraee, Hossein Golsorkh, Sajjad Mahdavi

**Affiliations:** School of Medicine, Shahid Beheshti University of Medical Sciences, Koodakyar Street, Tehran, Iran

**Keywords:** Hydromorphone, Cancer pain, Breakthrough pain, Adverse events, Analgesics, Opioids

## Abstract

**Background:**

Cancer pain significantly impacts individuals’ quality of life, with opioids being employed as the primary means for pain relief. Nevertheless, concerns persist regarding the adverse reactions and effectiveness of opioids such as morphine. Hydromorphone, recognized as a potent opioid, is a viable alternative for managing cancer-related pain. The goal of this systematic review and meta-analysis was to determine the effectiveness and safety characteristics of hydromorphone in comparison to other opioids, as well as different methods of administering this medication within the scope of cancer pain treatment.

**Methods:**

The PubMed, Embase, Cochrane Library, Scopus, and Web of Science databases were searched on December 25th, 2023. Following the PRISMA guidelines, a systematic investigation of databases was carried out, and suitable studies were chosen according to predetermined criteria (PICO framework). The meta-analyses were performed using a random-effects model.

**Results:**

This review included 18 RCTs with 2271 patients who compared hydromorphone with morphine, oxycodone, or fentanyl, as well as other types of hydromorphone. Hydromorphone demonstrated efficacy similar to that of morphine and oxycodone in reducing cancer pain intensity, decreasing additional analgesic consumption, and improving quality of life. However, morphine showed slight superiority over hydromorphone in reducing breakthrough pain. Adverse events were comparable between hydromorphone and morphine or oxycodone. Patient-controlled and clinician-controlled hydromorphone administration routes yielded similar outcomes.

**Conclusions:**

The outcomes of this study substantiate the efficacy of hydromorphone in the management of cancer-related pain, demonstrating similar levels of effectiveness and safety as morphine and oxycodone. These findings are consistent with prior comprehensive analyses, suggesting that hydromorphone is a feasible choice for alleviating cancer-associated pain. Additional investigations are warranted to determine its efficacy in distinct patient cohorts and for different modes of administration.

**Trial registration:**

Prospero registration ID: CRD42024517513. Link: https://www.crd.york.ac.uk/PROSPERO/#recordDetails.

**Supplementary Information:**

The online version contains supplementary material available at 10.1186/s12871-024-02638-y.

## Introduction

Cancer is associated with chronic pain that has deliberating effects on patients’ lives [1]. Approximately 44.5% of cancer patients experience pain, 30.6% of whom report pain that is moderate to severe [2]. The impact of pain on patient treatment adherence, survival rate, and quality of life has been substantiated [3]. Opioids serve as the primary pain relievers for managing cancer-related pain [4]. In regard to this topic, morphine is the most widely discussed opioid [5]. Nevertheless, morphine is linked to various issues, such as the risk of overdose, respiratory depression, and breakthrough pain [6–8]. breakthrough pain refers to an abrupt escalation of pain in individuals with chronic pain managed by analgesics [9]. Other options for the management of cancer-related pain include fentanyl, oxycodone, and hydromorphone [10].

Hydromorphone is a semisynthetic selective µ-opioid receptor agonist that was initially synthesized in Germany in 1921 and became a part of clinical practice by 1926 [11, 12]. It constitutes a powerful pharmaceutical opioid for treating acute pain of a moderate-to-severe nature as well as chronic pain of severe intensity in patients [13]. Compared with orally administered morphine, orally administered hydromorphone is 5 times more potent but has a similar side effect profile and stronger lipid solubility [12, 14]. Multiple recent clinical trials have focused on the utilization of hydromorphone for the management of cancer-related pain, as well as exploring various methods of administering this medication, making it crucial to thoroughly examine this topic systematically [15, 16]. 

We aimed to systematically review the literature to synthesize evidence regarding the efficacy and safety of hydromorphone for reducing cancer pain. To do so, we compared the intensity of cancer pain, rate of seeking additional analgesics, number of episodes of breakthrough pain, and quality of life between the hydromorphone arm and comparison arm, which included the morphine, oxycodone, and fentanyl arms. We further examined various forms of hydromorphone, such as patient-controlled and clinician-controlled therapy, sustained-release and immediate-release therapy, and subcutaneous and intravenous therapy.

## Methods

This systematic review and meta-analysis followed the guidelines outlined by the Preferred Reporting Items for Systematic Reviews and Meta-Analyses (PRISMA) [17]. The protocol for this review was submitted in advance to the International Prospective Register of Systematic Reviews (PROSPERO), with registration ID: CRD42024517513.

### Search strategy

On December 25, 2023, we conducted searches in PubMed, Embase, the Cochrane Library, Scopus, and Web of Science using keywords and MeSH terms synonymous with “Hydromorphone” and “Cancer”. Our search criteria did not impose any limitations regarding publication date or language. (Supplemental Content, Table 1)

### Study selection

The database search results were combined, and duplicate studies were eliminated using EndNote 20 software (Thomson Reuters, Toronto, ON, Canada). Two independent reviewers (S.M., S.O.) conducted two-phase title/abstract and full-text screening, while a third reviewer (M.A.) resolved any discrepancies between them. Studies that met the following eligibility criteria (based on the PICO framework) answered our PICO question—How effective is hydromorphone at alleviating cancer pain and how does its safety profile compare to alternative opioids in patients with cancer pain? —were included:

Participants: Patients diagnosed with cancer who had either of the following:

1. a mean pain intensity of at least 5 on the VAS or 4 on the NRS, or experienced breakthrough pain at least three times a day;
2. were receiving treatment with an oral opioid analgesic.

Intervention: Hydromorphone in any form.

Comparator: Placebo, substitute opioid, hydromorphone (different route and dose of administration), or another active control.

Outcomes: Cancer pain intensity, additional analgesic consumption, breakthrough pain, quality of life, and adverse events.

Study design: Randomized controlled trials (RCTs).

### Outcomes and data extraction

Our primary outcome was cancer pain intensity, which was measured by a visual analog scale (VAS), numerical rating scale (NRS), and brief pain inventory (BPI) at different time points in the studies.

Our secondary outcomes included additional analgesic consumption, breakthrough pain, quality of life, and adverse events.

Two reviewers (M.A., S.R.F.) collected relevant information from the chosen articles. This information encompassed various aspects, including authorship, publication year, country, study design, total number of participants, participants allocated to the hydromorphone group, those in the comparator group, age, gender, details of the comparator (drug, dosage, type), and hydromorphone (dosage, type), and discussed outcomes within the paper.

### Quality assessment

Two independent reviewers (H.G, S.M.) evaluated the risk of bias in the included studies using the Cochrane Collaboration’s tool for bias risk assessment [18], with oversight from a third reviewer (M.A.). The Cochrane tool examines domains such as random sequence generation, concealment of allocation to conditions, blinding of participants and personnel, blinding of outcome assessors, completeness of outcome data, selective reporting, and other biases. Each study was categorized based on bias risk: a low risk was assigned if no bias issues were detected, a high risk was assigned if bias issues were evident, and an unclear risk was assigned if there was insufficient information for assessment. A figure for risk of bias assessment was designed using the Risk of Bias Visualization tool (ROBVIS) [19].

### Certainty of evidence assessment

The GRADE method was used to assess the quality of the collected evidence which suggests four levels of certainty. High certainty indicates very high confidence that the true effect lies close to the estimate of the effect. Moderate certainty indicates moderate confidence in the effect estimate; the true effect is likely to be close to the estimate, but there is a possibility that it is substantially different. Low certainty indicates limited confidence in the effect estimate; the true effect may be substantially different from the estimate. Very low certainty indicates very little confidence in the effect estimate; the true effect is likely to be substantially different from the estimate. The evidence was evaluated based on five domains: risk of bias, inconsistency, indirectness, imprecision, and other considerations, which include publication bias, large effect, plausible confounding, and dose-response gradient. [20].

Randomized controlled trials were initially considered as high-certainty evidence. If any limitations were identified in one of these domains, the study’s certainty level was downgraded.

In the risk of bias domain, due to the low quality of major RCTs, we decided to downgrade the certainty of all outcomes once for serious risk of bias. Furthermore, if the heterogeneity of outcomes was greater than 50%, we decided to downgrade once for serious inconsistency. The evidence did not have serious indirectness because it was directly applicable to the PICO question. Due to the wide confidence interval of some results, which includes both clinically significant and non-significant effects, we decided to downgrade them once for serious imprecision. Because of the small number of studies for some outcomes, we could not evaluate publication bias and strongly suspected it. In the end, none of our results showed a large effect, plausible confounding, or dose-dependent gradient, which did not cause an upgrade in the level of evidence.

### Statistical analysis

A meta-analysis of all outcomes was carried out using STATA software version 17 (StataCorp LP, College Station, TX, USA), where the means and standard deviations were used as data for cancer pain intensity scores, additional analgesic usage, breakthrough pain, and quality of life. Cohen’s d was selected as the effect size for these four outcomes. The occurrence of adverse events, such as anorexia, constipation, diarrhea, dizziness, headache, nausea, pruritus, somnolence, urinary retention, and vomiting, was recorded, with the exponentiated risk ratio (RR) chosen as the effect size. The degree of heterogeneity was assessed using the I^2^ statistic, with an I^2^ value above 50% indicating substantial heterogeneity [21]. Due to significant variations in the methodologies employed by the studies, a random-effects model was utilized. Sensitivity analysis was performed using the leave-one-out method, and publication bias was evaluated using Egger’s regression test (a p value less than 0.05 indicating significant publication bias), and funnel plot symmetry was examined.

## Results

**Fig. 1:**
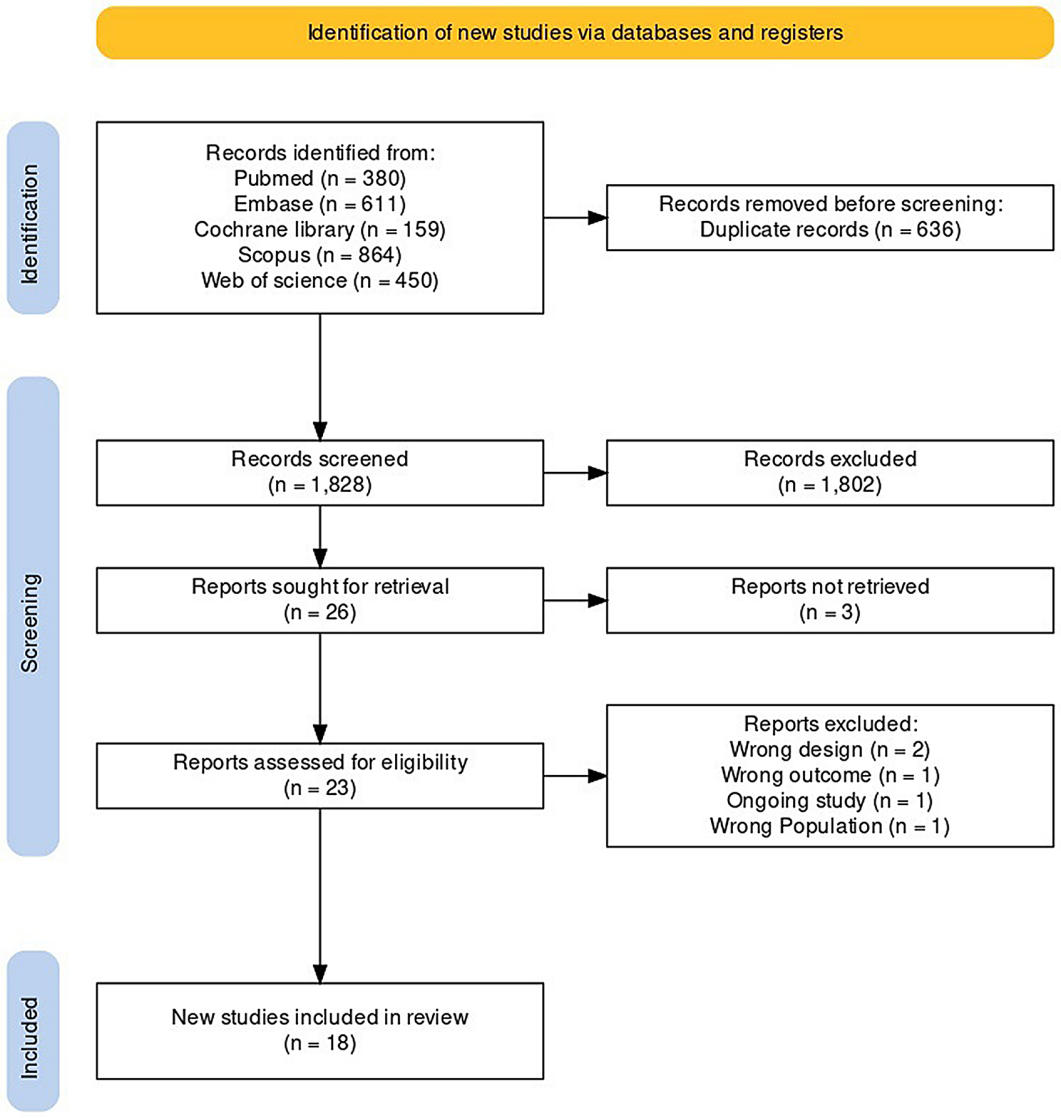
PRISMA flow diagram

Our online database search yielded 2464 papers, of which 1828 were chosen for title-abstract screening after the removal of 636 duplicates. Twenty-six papers were selected for full-text evaluation, 18 of which were included in our review (Fig. 1).

A total of 2271 patients were included in the RCTs. Seven articles compared hydromorphone with morphine [15, 16, 22–26], while five articles compared it with oxycodone [27–31]. One article compared hydromorphone with fentanyl [32], and five articles compared different types of hydromorphone. Among these, two compared patient-controlled versus clinician-controlled administration [33, 34], two compared sustained-release versus immediate-release formulations [35, 36], and one compared the subcutaneous route versus intravenous administration of hydromorphone [37]. Twelve RCTs were designed in parallel, while six were crossover trials. The publication dates of these studies ranged from 1988 to 2023. Various pain measurement scales were utilized across the studies: 10 studies employed the visual analog scale (VAS), six used the numerical rating scale (NRS), and two utilized the Brief Pain Inventory (BPI). The lowest and highest mean ages of the participants in the studies were 52.9 and 69.2 years, respectively. Details of the type, dosage, and route of administration of hydromorphone, comparison group, and other characteristics of the included clinical trials are available in Table 1.

**Table 1:** Study characteristics

Author (year)	Country	RCT design	Age, mean, y/ Sex	Number of patients Total, Hydromorphone, comparator	Hydromorphone type, dose	Comparison Drug; type, dose	Pain measure-ment scale	Secondary outcomes
Zeng [15] (2023)	China	Parallel	60.32/ 35 M; 22 W	60, 30, 30	PCSA, 0.3 mg/ml, 0.5 ml/h infusion speed	Morphine; PCSA 2 mg/ml, 0.5 ml/h infusion speed	NRS	AAC, breakthrough pain, QoL, AEs
Xiao [27] (2023)	China	Parallel	NA	256, 128, 128	PCSA	Oxycodone; SR	NRS	AAC, breakthrough pain, AEs
Yan [22] (2022)	China	Parallel	57/ 54 M; 44 W	98, 49, 49	SC, 10-20% of the TEOP24H	Morphine; SC, 10-20%of the TEOP24H	NRS	QoL, AEs
Lin [16] (2022)	China	Parallel	NA/ 51 M; 44 W	95,30/32, 33	IPCA, continuous & bolus infusion, 10-20%of the TEOP24H	Morphine; ER TEOP24H/2 × 75%	NRS	AAC, breakthrough pain, QoL, AEs
Banala [32] (2020)	United States	Parallel	52.9/ 37 M; 47 W	84, 42, 42	Intravenous, 1.5 mg	Fentanyl; Intranasal,100mcg	NRS	NA
Ma [23] (2020)	China	Parallel	60.43/ 152 M; 81 W	233, 121, 112	IT, mean 0.276 mg/day starting dose	Morphine; IT, mean 1.551 mg/day starting dose	VAS	Breakthrough pain, AEs
Lin [34] (2020)	China	Parallel	NA/ 123 M; 91 W	214, 106, 108	PCA, 10-20%of the TEOP24H	Hydromorphone; non-PCA,10-20%of the TEOP24H	NRS	AEs
Inoue [29] (2018)	Multicenter	Parallel	67.3/ 116 M; 56 W	181, 92, 89	IR, 4 mg/day + placebo	Oxycodone; IR, 10 mg/day + placebo	VAS	AEs
Inoue [28] (2017)	Multicenter	Parallel	69.2/ 108 M; 70 W	181, 88, 93	ER, 4 mg/day + ER placebo	Oxycodone; ER 10 mg/day + ER placebo	VAS	AEs
Yu [30] (2014)	China	Parallel	53.1/ 162 M; 86 W	260, 130, 130	ER, 8–32 mg	Oxycodone; CR, 10–40 mg	BPI	AAC, AEs
Hanna [24] (2008)	Multicenter	Parallel	59.8/ 98 M; 102 W	200, 99, 101	IR, for day 2–9 12–108 mg/day & SR for day 10–15	Morphine; IR, for 62–540 mg/day & SR	BPI	AAC, QoL, AEs
Moriarty [26] (1999)	NA	Crossover	NA/ 53 M; 47 W	100, NA, NA	CR, 4 mg	Morphine; CR, 30 mg	VAS	NA
Miller [25] (1999)	United Kingdom	Parallel	69/ 33 M; 41 W	77, 36, 41	Continuous SC infusion	Morphine; continuous SC infusion	VAS	AAC, AEs
Hagen [31] (1997)	Canada	Crossover	56/ 13 M; 18 W	44, 22, 22	CR, q12h	Oxycodone; CR q12h	VAS	AAC, AEs
Bruera [35] (1996)	Multicenter	Crossover	62/ 46 M; 49 W	95, 49, 46	SR, q12h + Placebo IRH q4h	Hydromorphone; IR, q4h + placebo SRH q12h	VAS	AAC
Hays [36] (1994)	Canada	Crossover	57.1/ 19 M; 26 W	48, NA, NA	CR, q12h	Hydromorphone; IR, q4h	VAS	AAC
Moulin [37] (1991)	Canada	Crossover	61/ 10 M; 10 W	20, NA, NA	SC	Hydromorphone; intravenous	VAS	Breakthrough pain
Bruera [33] (1988)	Canada	Crossover	54/ 10 M; 12 W	25, NA, NA	Patient-controlled SC infusion	Hydromorphone; continuous SC infusion	VAS	AEs

*Abbreviations* AAC: Additional analgesic consumption; AEs: Adverse events; BPI: Brief pain inventory; CR: Controlled-release; ER: Extended-release; IPCA: Intravenous patient-controlled analgesia; IR: Immediate-release; IT: Intrathecal; NA: Not available; NRS: Numerical rating scale; PCA: Patient-controlled analgesia; PCSA: Patient-controlled subcutaneous analgesia; QoL: Quality of life; SC: Subcutaneous; SR: Sustained-release; TEOP24h: Total equianalgesic over the previous 24 h; VAS: Visual analog scale

### Cancer pain

Thirteen controlled trials compared the effectiveness of hydromorphone in reducing cancer pain to that of morphine [15, 16, 22–26], oxycodone [27–31], and fentanyl [32].

### Hydromorphone versus morphine

Among the 7 trials comparing hydromorphone to morphine, Yan et al. found subcutaneous hydromorphone to be superior to subcutaneous morphine in reducing cancer pain after 24 h of treatment on an NRS among 98 participants (2.4 ± 0.4 versus 3.2 ± 0.5, p value < 0.001) [22]. Lin et al. 2022 reported that patient-controlled continuous hydromorphone with rescue bolus injections as well as rescue bolus injections of hydromorphone alone were superior to morphine after 6 days of treatment on an NRS among 95 participants (median NRS score of 2.0 versus 2.0 versus 3.0) [16]. More recently, Zeng et al. reported that patient-controlled subcutaneous hydromorphone was superior to morphine after 30 min of treatment on an NRS among 57 participants (3.9 ± 2.6 vs. 5.3 ± 2.1, p value = 0.035) but yielded comparable pain scores by the end of treatment (3.2 ± 1.8 versus 3.2 ± 1.5) [15]. The other 4 trials [23–26], however, found hydromorphone to be equally effective as morphine in reducing cancer pain scores among a pooled 576 participants.

**Fig. 2:**
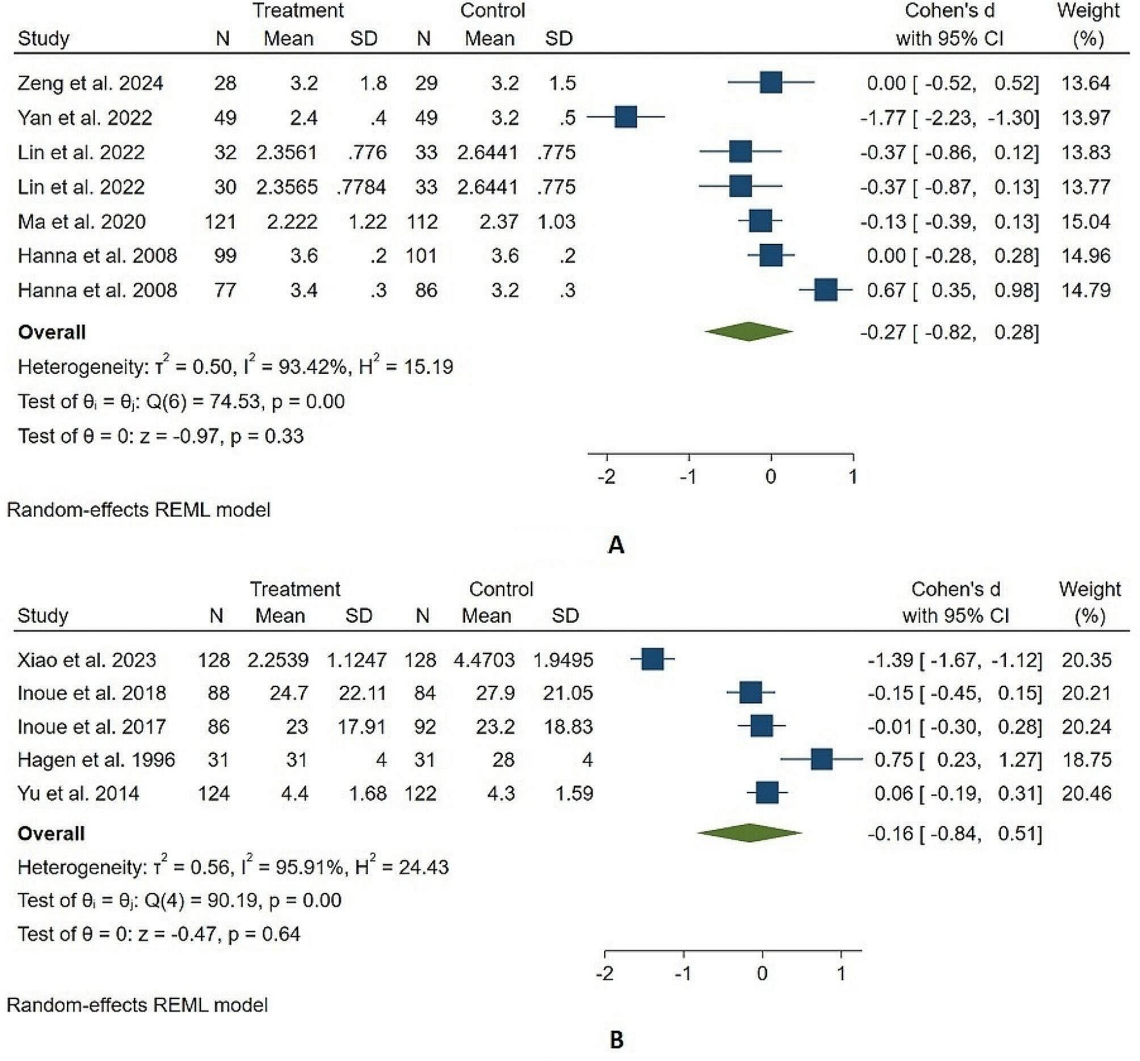
The forest plot illustrates the comparison of the reduction in cancer pain between the hydromorphone group and the morphine group in the studies (**A**), and between the hydromorphone group and the oxycodone group in the studies (**B**)

Five of these 7 trials had sufficient data to perform a meta-analysis, the pooled results of which showed a similar reduction in cancer pain scores between 436 hydromorphone-treated participants and 443 morphine-treated participants (Cohen’s d, (95% CI); -0.27, (-0.82, 0.28)), although there was significant heterogeneity (I^2^ = 93.42%, Fig. 2A) and a very low certainty GRADE rating (Table 2). Sensitivity analysis showed our findings to be stable (Supplemental Content, Fig. 1), and no publication bias was detected using Egger’s regression test or funnel plot symmetry (Supplemental Content, Fig. 2). The two trials not included in the meta-analysis revealed hydromorphone to be noninferior to morphine in reducing cancer pain scores [25, 26], which is in accordance with our meta-analysis and further supports our finding of its comparable analgesic efficacy for cancer pain.

**Table 2:** Grade evidence

Certainty assessment							№ of patients		Effect		Certainty
№ of studies	Study design	Risk of bias	Inconsistency	Indirectness	Imprecision	Other considerations	Hydromorphone	Control	Relative	Absolute (95% CI)	
Cancer pain intensity (vs. morphine)											
7	randomized trials	serious^a^	serious^b^	not serious	serious^c^	none	417	416	-	SMD − **0.27** (-0.82 to + 0.28)	⨁◯◯◯ Very low
**Cancer pain intensity (vs. oxycodone)**											
5	randomized trials	serious^a^	serious^b^	not serious	serious^c^	none	460	462	-	SMD − **0.16** (-0.84 to + 0.51)	⨁◯◯◯ Very low
**Additional analgesic consumption (vs. morphine)**											
4	randomized trials	serious^a^	not serious	not serious	serious^c^	Publication bias^d^	211	214	-	SMD + **0.13** (-0.11 to + 0.36)	⨁◯◯◯ Very low
**Breakthrough pain (vs. morphine)**											
3	randomized trials	serious^a^	not serious	not serious	not serious	Publication bias^d^	183	175	-	SMD + **0.19** (0 to + 0.39)	⨁⨁◯◯ Low
**Quality of life (vs. morphine)**											
4	randomized trials	serious^a^	serious^b^	not serious	serious^c^	Publication bias^d^	210	213	-	SMD − **0.03** (-0.51 to + 0.45)	⨁◯◯◯ Very low
**Adverse events (vs. morphine)**											
6	randomized trials	serious^a^	not serious	not serious	serious^c^	Publication bias^d^	367	366	log RR **-0.06**		⨁◯◯◯ Very low
**Adverse events (vs. oxycodone)**											
5	randomized trials	serious^a^	not serious	not serious	serious^c^	Publication bias^d^	460	462	log RR **+ 0.04**		⨁◯◯◯ Very low

**CI**: confidence interval; **SMD**: standardized mean difference; **RR**: relative risk

*Explanations*

a. Due to low quality of RCTs, we decided to downgrade once for serious risk of bias

b. Due to a heterogeneity greater than 50%, we decided to downgrade once for serious inconsistency

c. Due to the wide confidence interval, which includes both clinically significant and non-significant effects, we decided to downgrade once for serious imprecision of the effect estimate

d. Due to potential publication bias, we decided to downgrade once for strong susceptibility to publication bias

### Hydromorphone versus oxycodone

**Fig. 3:**
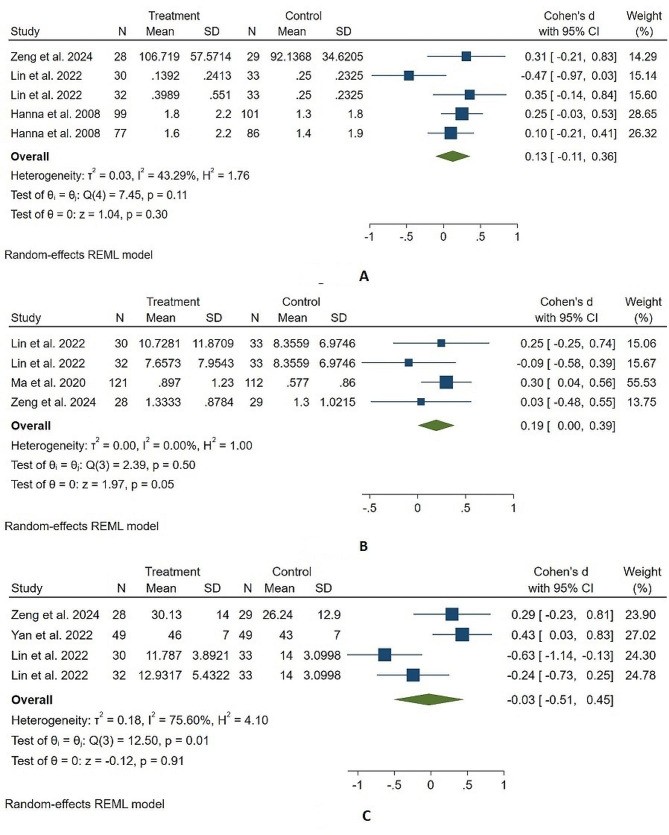
The forest plot illustrates the comparison of additional analgesic consumption (**A**), breakthrough pain (**B**), and quality of life (**C**) between the hydromorphone group and the morphine group in the studies

**Fig. 4:**
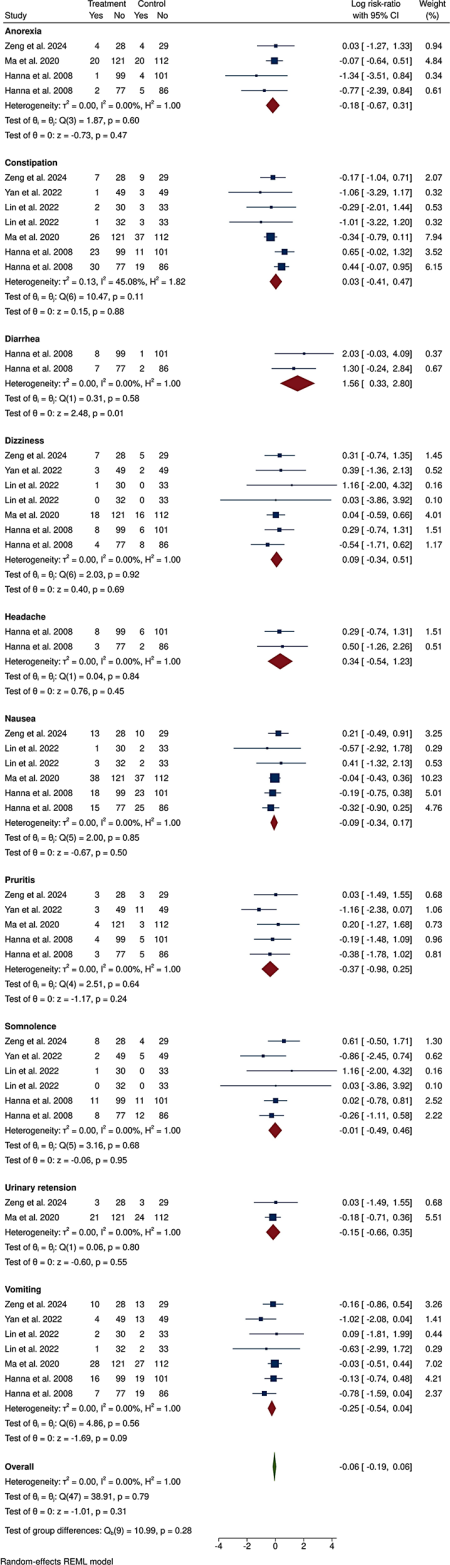
The forest plot illustrates the comparison of adverse events between the hydromorphone group and the morphine group in the studies

Among the 5 trials comparing hydromorphone to oxycodone, Xiao et al. found patient-controlled subcutaneous hydromorphone to be superior to oxycodone after 12 h of treatment on an NRS among 256 participants (median (95% CI): 2.5 (1.4–2.9) versus 4.4 (3.2–5.8); p value < 0.001) [27]; however, the other 4 trials found that hydromorphone has an analgesic profile similar to that of oxycodone among a pooled sample of 531 participants. The meta-analysis of these 5 controlled trials showed that hydromorphone is as effective as oxycodone in reducing cancer pain (Cohen’s d, 95% CI; -0.16 (-0.84, 0.51)), albeit with significant heterogeneity (I^2^ = 95.91%, Fig. 2B) and a very low certainty GRADE rating (Table 2). Sensitivity analysis showed our findings to be robust (Supplemental Content, Fig. 3), and no publication bias was evident according to Egger’s regression test or funnel plot symmetry (Supplemental Content, Fig. 4).

### Hydromorphone versus fentanyl

Banala et al. [32] compared clinician-controlled intravenous hydromorphone with intranasal fentanyl 4 h after treatment on an NRS among 82 participants and found that hydromorphone was noninferior to intranasal fentanyl, with a similar median pain score after 1 h of treatment (3.5 vs. 3.0).

## Additional analgesic consumption

Seven of the included controlled trials compared additional analgesics used by cancer patients receiving hydromorphone with those used by patients receiving morphine [15, 16, 24, 25] or oxycodone [27, 30, 31].

### Hydromorphone versus morphine

Among the 4 controlled trials comparing additional analgesic consumption in hydromorphone-treated patients to that in morphine-treated patients, Miller et al. reported that patients receiving continuous subcutaneous hydromorphone infusion were almost twice as likely (log RR, 95% CI, p value; 2.2, (1.1–4.6), 0.03) to require additional analgesics in the first 24 h of treatment, yet a similar rate of additional analgesic consumption was achieved 24–72 h after treatment initiation (log RR, 95% CI, p value; 0.8, (0.4–1.8), 0.5) [25]. The other 3 trials found patients receiving hydromorphone require the same dose of additional analgesics as morphine-treated patients, with the recent study by Zeng et al. finding a similar rate of 0–24 (median of 117 versus 114, p value = 0.191), 24–48 (median of 102.6 versus 87, p value = 0.296), and 48–72 (median of 101.4 versus 87, p value = 0.716) hours after treatment with patient-controlled subcutaneous hydromorphone [15], while Lin et al. 2022. found the rate to be similar after 6 days of treatment with both patient-controlled continuous infusion hydromorphone (median of 0.26 versus 0.25, p value = 1.0) as well as bolus-only infusion hydromorphone (median of 0.10 versus 0.25, p value = 0.261) [16] and Hanna et al. found the rate to be similar after 2–9 days of immediate-release hydromorphone therapy (mean of 1.8 ± 2.2 versus 1.3 ± 1.8) as well as after 10–15 days of sustained-release hydromorphone therapy (mean of 1.6 ± 2.2 versus 1.4 ± 1.9) [24].

**Fig. 5:**
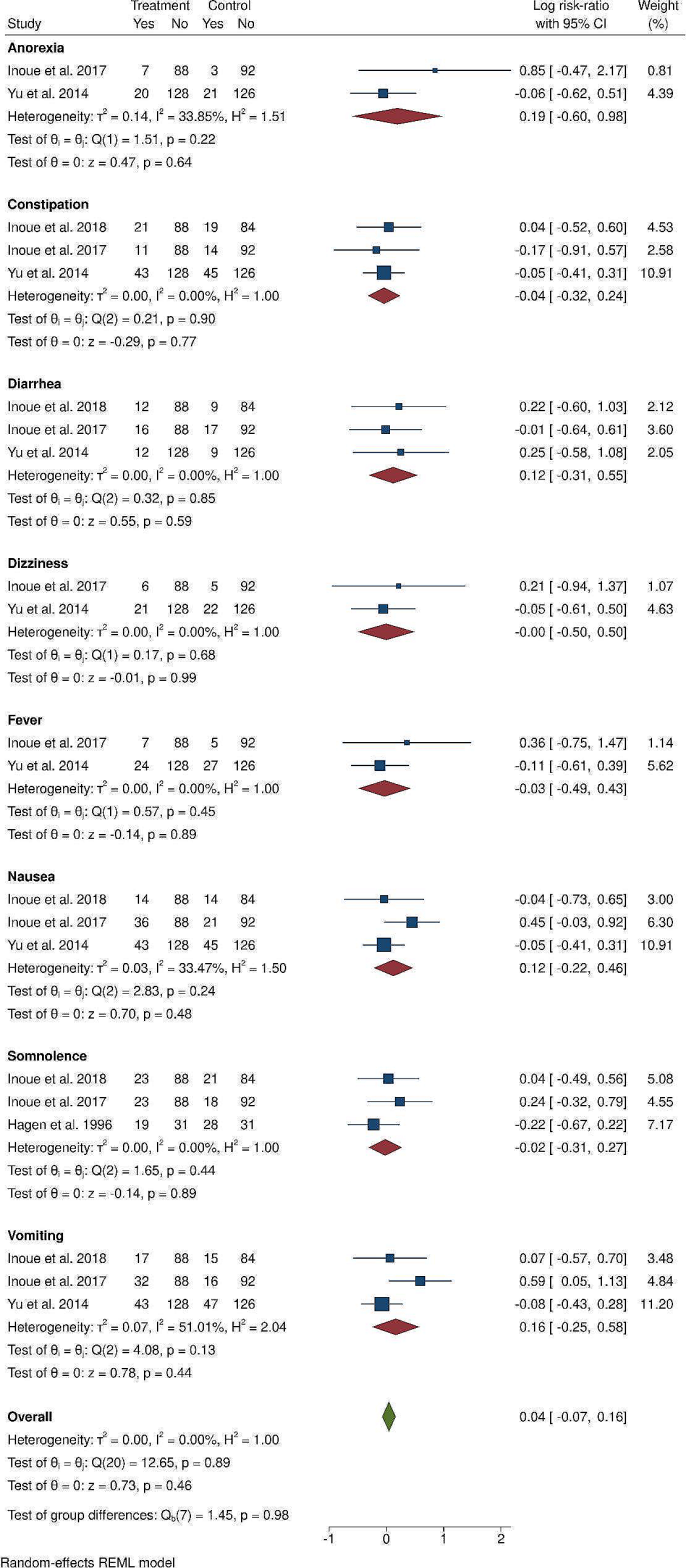
The forest plot illustrates the comparison of adverse events between the hydromorphone group and the oxycodone group in the studies

Three of the 4 trials had sufficient data to be included in our meta-analysis and showed a nonsignificant increase in additional analgesic consumption in hydromorphone-treated patients compared to that in morphine-treated patients (Cohen’s d, 95% CI; 0.13, (-0.11, 0.36)), with low heterogeneity (I^2^ = 43.29%, Fig. 3A) and a very low certainty GRADE rating (Table 2). The sensitivity analysis showed our findings to be stable (Supplemental Content, Fig. 5), and publication bias was not assessed due to the limited number of studies included in our meta-analysis.

### Hydromorphone versus oxycodone

Three controlled trials compared additional analgesic consumption between hydromorphone-treated patients and oxycodone-treated patients. Among these, the study by Hagen et al. was the first to compare controlled-release hydromorphone with controlled-release oxycodone in a crossover study of 31 patients who were treated for 7 days and reported similar mean daily rescue analgesic consumption between patients receiving controlled-release hydromorphone and those receiving controlled-release oxycodone (1.6 versus 1.4) [31]. Recently, Yu et al. reported that the mean number of additional analgesics consumed by 36 patients treated with extended-release hydromorphone was similar to that consumed by 40 patients treated with controlled-release oxycodone (24.2 versus 29.3) during a 28-day maintenance phase [30]. Recently, Xiao et al. reported similar additional analgesic consumption in 128 patients receiving patient-controlled subcutaneous hydromorphone compared to 128 patients receiving sustained-release oxycodone [27]. Overall, hydromorphone and oxycodone achieved a similar rate of additional analgesic consumption among a pooled 363 cancer patients.

## Breakthrough pain

Four controlled trials compared the number of breakthrough pains throughout hydromorphone treatment with that of morphine [15, 16, 23] or oxycodone [27]. Furthermore, all four trials noted a reduced frequency of breakthrough pain during hydromorphone therapy.

### Hydromorphone versus morphine

**Fig. 6:**
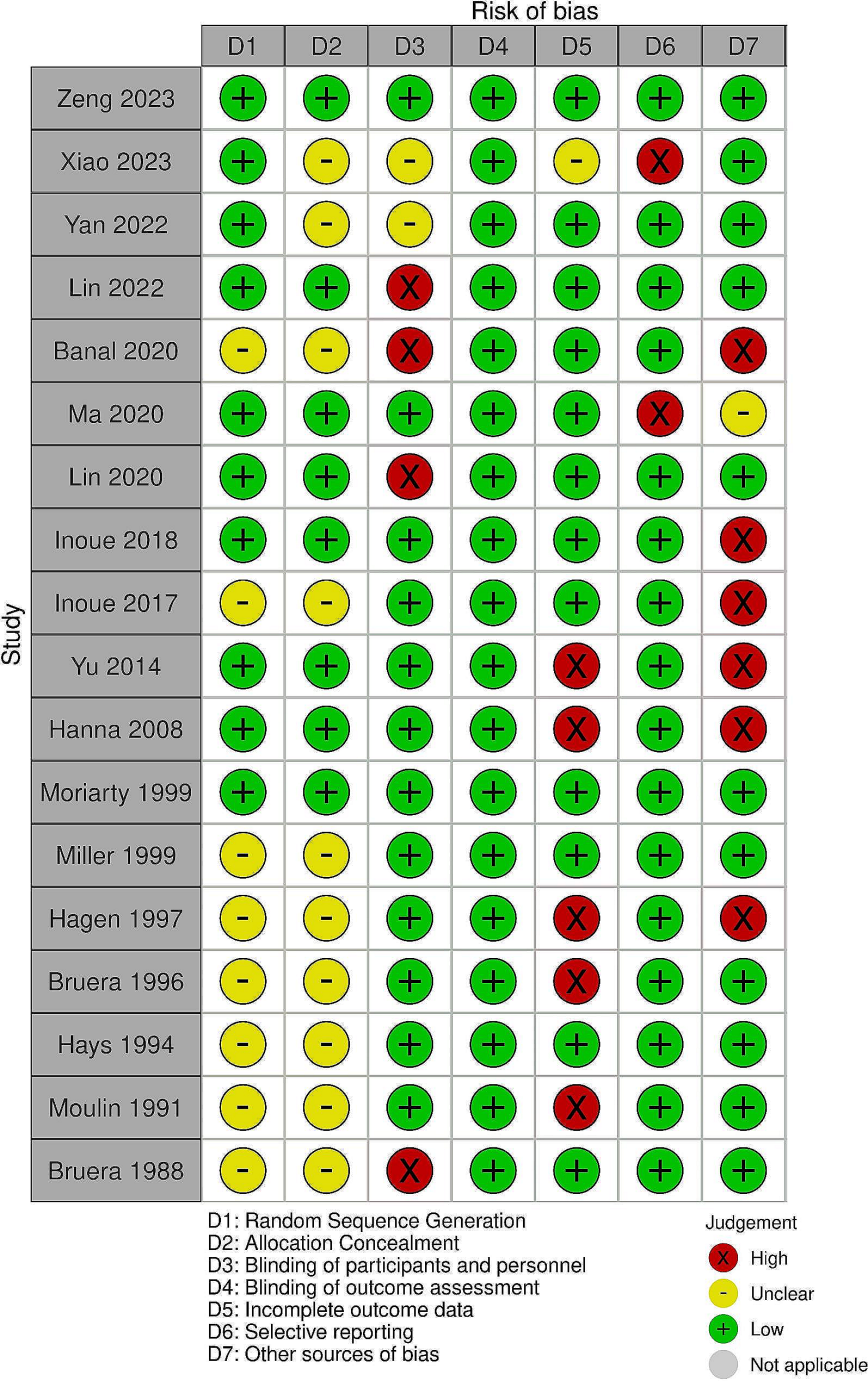
Quality assessment of studies based on Cochrane’s tool

Zeng et al. compared the frequency of breakthrough pain in 29 patient-controlled subcutaneous hydromorphone-treated patients with that in 28 morphine-treated patients during 0–24 h of treatment (mean of 1.8 ± 1.1 versus 2.1 ± 1.3), 24–48 h of treatment (1.3 ± 0.7 versus 1.0 ± 0.5), and 48–72 h of treatment (0.9 ± 0.5 versus 0.8 ± 0.5) and found no significant difference between the two [15]. Lin et al. (2022) compared the frequency of breakthrough pain in 30 patient-controlled continuous plus bolus infusion-treated patients or 32 bolus-only infusions of hydromorphone-treated patients and reported a similar frequency of breakthrough pain to that in morphine-treated patients (median of 6.5 versus 8.5 versus 8.0, p value = 0.811); however, they did not observe a reduced duration of breakthrough pain in patient-controlled continuous plus bolus infusion-treated patients compared to that in morphine-treated patients (median of 11.76 versus 16.0, p value = 0.025) but not in bolus-only infusions of hydromorphone-treated patients. Ma et al. compared the incidence of breakthrough pain in 121 patient-controlled intrathecal hydromorphone-treated patients with that in 112 morphine-treated patients and reported a similar rate after 12 weeks of treatment (mean of 0.90 ± 1.23 versus 0.58 ± 0.86, p value = 0.195) as well as throughout the treatment [23]. Our meta-analysis of these 3 trials revealed a slight yet significant increase in the frequency of breakthrough pain in hydromorphone-treated patients compared to that in morphine-treated patients (Cohen’s d, 95% CI; 0.19, 0.0-0.39) with low heterogeneity (I^2^ = 0%, Fig. 3B) and a low certainty GRADE rating (Table 2); however, our sensitivity analysis revealed a lack of stability (Supplemental Content, Fig. 6).

### Hydromorphone versus oxycodone

Xiao et al. were the only study to compare patient-controlled subcutaneous hydromorphone to oral oxycodone tablets and found that 128 patients receiving hydromorphone experienced fewer incidences of breakthrough pain than did 128 patients receiving morphine (mean of 121 ± 28 versus 186 ± 31; p value < 0.001; Cohen’s d (95% CI): -2.20 (-2.51, -1.89).

Quality of life.

Five of the included studies compared the quality of life of cancer patients receiving hydromorphone with that of cancer patients receiving morphine [15, 16, 22, 24] or oxycodone [27].

### Hydromorphone versus morphine

Zeng et al. recently compared quality of life using the brief pain inventory (BPI) after 24 and 72 h of treatment with patient-controlled subcutaneous hydromorphone in 28 patients and 29 patients receiving morphine and found no significant difference between the two in the overall score (mean of 34.93 ± 15.3 versus 30.47 ± 12.8 at 24 h, p value = 0.438; mean of 30.13 ± 14 versus 26.24 ± 12.9 at 72 h, p value = 0.288) [15]. Improvements in sleep, mood, enjoyment of life, general activity, walking ability, normal work, and relationships with other persons were also noted after 24 and 72 h of hydromorphone therapy. Yan et al. compared the quality of life after 24 h of subcutaneous hydromorphone therapy in 49 cancer patients with 49 cancer patients receiving morphine and reported comparable quality of life scores after treatment (mean of 46 ± 7 versus 43 ± 7; p value = 0.109) [22]. Lin et al. (2022) compared quality of life using the Edmonton Symptom Assessment System (ESAS) after and during 6 days of treatment in 32 patients receiving patient-controlled continuous infusion hydromorphone, 30 patients receiving bolus-only infusions of hydromorphone, and 33 patients receiving morphine and found no difference in the overall score after 3 days (median of 13.5 versus 15.5 versus 16.0) or 6 days of treatment (median of 14.0 versus 12.5 versus 14.0) [16]. No significant differences in tiredness, nausea, depression, anxiety, drowsiness, anorexia, well-being, itching, or dyspnea between hydromorphone-treated and morphine-treated patients were observed in their study; however, the pain score on the ESAS was lower in the hydromorphone-treated patients than in the morphine-treated patients after 3 days (median of 2.0 versus 2.0 versus 4.0) or 6 days (median of 2.0 versus 2.0 versus 3.0) of treatment. The report by Hanna et al. was the first to compare the quality of life between immediate-release and sustained-release hydromorphone therapy with immediate-release and sustained-release morphine therapy using the BPI and revealed a similar improvement in quality of life between immediate-release treated hydromorphone- and morphine-treated patients and sustained-release hydromorphone- and morphine-treated patients in terms of the overall score as well as in each item, except for normal work, which showed a more pronounced improvement in the 99 immediate-release hydromorphone-treated patients than in the 101 immediate-release morphine-treated patients (p value = 0.03) [24]. Excluding the study by Hanna et al., the other 3 trials comparing hydromorphone to morphine at the end of treatment were included in our meta-analysis and showed no significant difference between the quality of life of hydromorphone-treated patients and that of morphine-treated patients (Cohen’s d (95% CI): -0.03 (-0.51, 0.45), with significant heterogeneity (I^2^ = 75.60%, Fig. 3C) and a very low certainty GRADE rating (Table 2). Sensitivity analysis also showed our findings to be robust (Supplemental Content, Fig. 7).

### Hydromorphone versus oxycodone

Xiao et al. compared the effectiveness of oxycodone in improving quality of life in 128 patients receiving patient-controlled subcutaneous hydromorphone and 128 patients receiving oral oxycodone and reported a similar significant improvement [27].

## Adverse events

The adverse events of hydromorphone therapy in cancer patients were studied in 11 controlled trials, with 6 comparing it to morphine [15, 16, 22–25] and 5 comparing it to oxycodone [27–31]. Among the hydromorphone-treated patients, anorexia was experienced by 54 of 541 participants across 5 studies [15, 23, 24, 28, 30], nausea was experienced by 181 of 691 participants across 7 studies [15, 16, 23, 24, 28–30], constipation was experienced by 165 of 740 participants across 8 studies [15, 16, 22–24, 28–30], vomiting was experienced by 160 of 740 participants across 8 studies [15, 16, 22–24, 28–30], diarrhea was experienced by 55 of 480 participants across 4 studies [24, 28–30], dizziness was experienced by 68 of 652 participants across 7 studies [15, 16, 23, 24, 28–30], fever was experienced by 31 of 216 participants in 2 studies [28, 30], headache was experienced by 11 of 176 participants in 1 study [24], pruritis was experienced by 17 of 374 participants across 4 studies [15, 22–24], somnolence was experienced by 95 of 552 participants across 7 studies [15, 16, 22, 24, 28, 29, 31], and urinary retention was experienced by 24 of 149 participants in 2 studies [15, 23]. Other adverse events were reported by less than 5% of participants.

### Hydromorphone versus morphine

Among the 6 studies comparing adverse events in hydromorphone-treated patients with those in morphine-treated patients, all 6 reported a similar rate of adverse events, except for the study by Hanna et al., who reported a greater rate of diarrhea in hydromorphone-treated patients (15/176, 8.5%) than in morphine-treated patients (3/187, 1.6%) [24].

Five of these studies had sufficient data to be included in our meta-analysis, which revealed no significant difference in the relative risk of anorexia, constipation, diarrhea, dizziness, headache, nausea, pruritus, somnolence, urinary retention, or vomiting, with no significant heterogeneity in any of the meta-analyses (I^2^ = 0%), except for the incidence of constipation (I^2^ = 45.08%, Fig. 4). Overall, a similar risk ratio of adverse events between morphine- and hydromorphone-treated patients was also observed (log RR (95% CI): -0.06 (-0.19, 0.06)), with low heterogeneity (I^2^ = 0%, Fig. 4) and a very low certainty GRADE rating (Table 2). The study by Miller et al., which was not included in the meta-analysis, also revealed a similar rate of adverse events between the two groups [25].

### Hydromorphone versus oxycodone

Among the 5 studies comparing hydromorphone to oxycodone, all 5 reported a similar rate of adverse events, except for one study by Inoue et al., who reported a greater rate of vomiting in hydromorphone-treated patients (32/88, 36.4%) than in morphine-treated patients (16/92, 17.4%) [28].

Four of the included studies had sufficient data to be included in our meta-analysis, which revealed no significant difference in the risk ratio of anorexia, constipation, diarrhea, dizziness, fever, nausea, somnolence, or vomiting, with low heterogeneity (I^2^ < 50%) in every adverse event excluding vomiting (I^2^ = 51.01%, Fig. 5). Overall, a similar risk ratio of adverse events between oxycodone- and hydromorphone-treated patients was achieved (log RR (95% CI): 0.04 (-0.07, 0.16)), with low heterogeneity (I^2^ = 0%, Fig. 5) and a very low certainty GRADE rating (Table 2). The study by Xiao et al., which was not included in our meta-analysis, also revealed a similar rate of adverse events between the two groups [27].

## Comparison of the different routes of administration of hydromorphone

Among the included studies, 2 controlled trials compared the effectiveness of patient-controlled and clinician-controlled hydromorphone therapy. In the initial crossover study by Bruera et al.1988, 22 patients with severe cancer pain were treated for 3 days with either patient-controlled subcutaneous or continuous subcutaneous infusions of hydromorphone [33], while in the recent study by Lin et al. 2020. A total of 214 patients with severe cancer pain were treated for 24 h, with 108 receiving clinician-controlled intravenous hydromorphone and 106 receiving patient-controlled intravenous hydromorphone [34]. Pain and adverse events were measured using a visual analog scale [33] or the ESAS [34] in both studies, and pooled comparisons between the two groups upon completion of treatment are available in (Supplemental Content, Table 2). The total concentration of hydromorphone received was similar between the two groups in both studies. The time to successful titration was also compared in the study by Lin et al., who reported that the time to successful titration was significantly shorter in 106 patient-controlled patients than in 108 clinician-controlled patients (median (95% CI): 0.5 h (0.25, 0.50) versus 0.79 (0.50, 1.42); p value = 0.001).

To date, only two clinical trials have directly compared sustained-release and immediate-release hydromorphone therapy: the crossover study by Bruera et al. (1996) with 95 participants [35] and the two-way crossover study by Hays et al. with 44 participants [36]. Both studies reported similar VAS pain scores after treatment and comparable daily additional analgesic consumption during treatment (Supplemental Content, Table 3). Furthermore, both studies reported similar nausea and sedation intensity scores, as did the study by Hays et al., who also reported a similar incidence of adverse events between the two groups [36].

Moulin et al. compared continuous subcutaneous hydromorphone infusion with continuous intravenous hydromorphone infusion over 48 h of treatment using a VAS among 15 participants and found no significant differences between the routes. Additionally, the mean number of infusions after breakthrough pain did not differ significantly between the subcutaneous and intravenous routes of administration (4.8 versus 5.3) [37].

### Quality assessment

The assessment of the risk of bias was conducted using the Cochrane Collaboration tool, revealing that the majority of the studies included had a high risk of bias in at least one domain. We judged a high risk of bias in the “other bias” domain as the most common cause of bias, as six of the studies were funded by pharmaceutical companies. Additionally, five studies were rated at high risk of incomplete outcome data bias due to more than 10% dropout, and the blinding of participants and personnel in four studies was judged at high risk of bias because they were considered open-label studies.

Overall, the quality assessment indicated low quality in most of the included randomized controlled trials (RCTs). (Fig. 6)

## Discussion

This systematic review and meta-analysis aimed to assess the effectiveness of hydromorphone in reducing pain among oncology patients and to compare its efficacy to that of other opioids. The review revealed that all included investigations reported a significant reduction in pain among patients, and the meta-analyses indicated a similar efficacy to that of morphine and oxycodone. Our analyses also demonstrated the similar efficacy of hydromorphone to that of morphine and oxycodone in terms of reducing additional analgesic consumption and increasing quality of life. In addition, the literature suggests that improvements in sleep quality are also similar to those associated with other opioids. Hydromorphone, however, was not similar to other opioids in its ability to reduce breakthrough pain; our analysis showed that morphine had a slight advantage over hydromorphone, and hydromorphone was noted to be more effective than oxycodone in the included study that compared the two. Additionally, one report suggested that hydromorphone requires less time to be successfully titrated than morphine. No significant difference was noted in the analgesic effect of hydromorphone when patient-controlled versus clinician-controlled administration routes were compared or when sustained-release and immediate-release administrations were contrasted.

The results we obtained are congruent with those of other systematic reviews exploring the analgesic effects of hydromorphone and other opioids in cancer patients. In 2011, Pigni et al. [38] conducted a systematic review of 13 clinical trials in the literature, regardless of randomization or the presence of a control group. Although the heterogeneity of the studies did not allow for a meta-analysis, their review of the literature suggested that the efficacy and tolerability of hydromorphone for the management of moderate to severe cancer pain are on par with those of oxycodone and morphine. However, there are insufficient data indicating its superiority or inferiority to morphine as the primary option for cancer pain management. Another systematic study by Caraceni et al. [39] reviewing the evidence supporting oral morphine as the first-choice opioid for treating cancer pain also revealed that morphine, hydromorphone, oxycodone, and methadone offer similar pain relief with a similar pattern of toxicity. The same conclusion was reached by King et al. [40], who aimed to systematically review the use of oxycodone in the management of cancer pain. In addition, a notable study by King et al. [41] assessing the use of opioids for cancer patients with renal impairment revealed clinical experience and some published retrospective data suggesting that hydromorphone may be safer than morphine in treating renal impairment. In a 2016 update of this review, Sande et al. [42] speculated that the low protein binding, low molecular weight, and low volume of distribution of hydromorphone reduce its accumulation, and by extension, the rate of adverse effects. Finally, the most recent systematic review assessing hydromorphone for cancer pain was a 2021 Cochrane review [43] encompassing 8 randomized controlled trials. This review revealed a high level of uncertainty in evidence weighing the advantages and drawbacks of hydromorphone over other opioids and concluded that insufficient evidence exists to support or refute the use of hydromorphone over other opioids.

In addition to the outcomes regarding the safety and efficacy of hydromorphone, the noninferiority of patient-controlled analgesia (PCA) to traditional administration methods is noteworthy and consistent with the relevant literature. In a systematic review assessing opioid administration via PCA in cancer pain, Nijland et al. [44] noted that PCA opioid use was safe and useful in cancer pain management, with the caveat that most of the included studies were of low quality. As PCA is the fourth step on the analgesic ladder for cancer pain treatment [45], it is imperative that additional high-quality studies be conducted to assess its safety and efficacy so that more definite conclusions can be drawn. The similarity of sustained-release hydromorphone to the immediate-release version is also notable, as using the sustained-release version translates to more comfortable, once-daily dosing instead of around-the-clock analgesic use [46]. Further high-quality research is needed to confirm this observation.

### Strengths and limitations

Our analyses are bolstered by the low heterogeneity found in outcomes such as additional analgesic consumption and the rate of adverse events. Moreover, low publication bias and high sensitivity in most of the outcomes add to the validity of the meta-analyses. Nevertheless, our study is constrained by several limitations. First and foremost, significant heterogeneity was present in the analyses assessing the analgesic effect of hydromorphone and its effect on the quality of life of the patients, thus warranting caution when interpreting the results. Second, many of the studies included in our review were judged to be of low quality. Additionally, the number of included studies and patients in many of the assessed endpoints was low. As such, we recommend that future high-quality studies with larger sample sizes be conducted so that these endpoints can be assessed more effectively.

## Conclusion

In conclusion, this review substantiates the safety and efficacy of hydromorphone in the management of cancer pain. Evidence suggests that hydromorphone is similar to morphine and oxycodone in providing pain relief and increasing quality of life, although the studies conducted in this regard are heterogeneous. Furthermore, it was demonstrated that patients who consumed hydromorphone had similar additional analgesic consumption and rates of adverse events to those who consumed morphine and oxycodone. However, morphine appears to have a slight advantage over hydromorphone in suppressing breakthrough pain. Future studies are needed to increase the quality of evidence regarding hydromorphone use in cancer pain treatment and to explore aspects such as sustained-release administration and patient-controlled analgesia.

### Electronic supplementary material

Below is the link to the electronic supplementary material.

Supplementary Material 1

## Data Availability

No datasets were generated or analysed during the current study.
