# Supplementary material for: Efficacy and safety of hydromorphone for cancer pain: a systematic review and meta-analysis
Source: BMC Anesthesiol. 2024 Aug 9;24:283. doi: 10.1186/s12871-024-02638-y (PMC11312680; doi:10.1186/s12871-024-02638-y)
Supplement: Supplementary file 1 — Supplementary Material 1 [file 12871_2024_2638_MOESM1_ESM.docx]

**Supplemental materials**

**Efficacy and Safety of Hydromorphone for Cancer Pain: A Systematic Review and Meta-analysis of Controlled Trials**

Mohammadreza Alinejadfard, Shahryar Rajai Firouzabadi, Ida Mohammadi, Soroush Oraee, Hossein Golsorkh, Sajad Mahdavi

**Supplemental Table 1. Search strategy**

**PubMed**

| **Line** | **Search Term** |
| --- | --- |
| 1 | ((hydromorphone [Title/Abstract]) OR (dihydromorphinone [Title/Abstract]) OR (palladone [Title/Abstract]) OR (Laudacon [Title/Abstract]) OR (Dilaudid [Title/Abstract]) OR (Hydromorphone Hydrochloride [Title/Abstract])) |
| 2 | ((cancer [Title/Abstract]) OR (tumor-related pain [Title/Abstract]) OR (tumor-associated pain [Title/Abstract]) OR (neoplasm-related pain [Title/Abstract]) OR (neoplasm-associated pain [Title/Abstract]) OR (oncological pain [Title/Abstract])) |
| 3 | 1 AND 2 |

**Embase**

| **Line** | **Search Term** |
| --- | --- |
| 1 | (hydromorphone:ti,ab,kw OR dihydromorphinone:ti,ab,kw OR 'hydromorphone hydrochloride':ti,ab,kw OR dilaudid:ti,ab,kw OR palladone:ti,ab,kw OR laudacon:ti,ab,kw) |
| 2 | (cancer:ti,ab,kw OR 'tumor-related pain':ti,ab,kw OR 'tumor-associated pain':ti,ab,kw OR 'neoplasm-related pain':ti,ab,kw OR 'neoplasm-associated pain':ti,ab,kw OR 'oncological pain':ti,ab,kw) |
| 3 | 1 AND 2 |

**Cochrane library**

| **Line** | **Search Term** |
| --- | --- |
| 1 | ("hydromorphone"):ti,ab,kw OR ("dihydromorphinone"):ti,ab,kw OR ("hydromorphone hydrochloride"):ti,ab,kw OR ("Dilaudid"):ti,ab,kw OR ("Palladone"):ti,ab,kw OR ("laudacon"):ti,ab,kw |
| 2 | ("cancer"):ti,ab,kw OR ("tumor-related pain"):ti,ab,kw OR ("tumor-associated pain"):ti,ab,kw OR ("neoplasm-related pain"):ti,ab,kw OR ("neoplasm-associated pain"):ti,ab,kw OR ("oncological pain"):ti,ab,kw |
| 3 | 1 AND 2 |

**Scopus**

| **Line** | **Search Term** |
| --- | --- |
| 1 | ( ( TITLE-ABS-KEY ( hydromorphone ) OR TITLE-ABS-KEY ( dihydromorphinone ) OR TITLE-ABS-KEY ( palladone ) OR TITLE-ABS-KEY ( laudacon ) OR TITLE-ABS-KEY ( dilaudid ) OR TITLE-ABS-KEY ( hydromorphone AND hydrochloride ) ) ) |
| 2 | ( ( TITLE-ABS-KEY ( cancer ) OR TITLE-ABS-KEY ( tumor-related AND pain ) OR TITLE-ABS-KEY ( tumor-associated AND pain ) OR TITLE-ABS-KEY ( neoplasm-related AND pain ) OR TITLE-ABS-KEY ( neoplasm-associated AND pain ) OR TITLE-ABS-KEY ( oncological AND pain ) ) ) |
| 3 | 1 AND 2 |

**Web of Science**

| **Line** | **Search Term** |
| --- | --- |
| 1 | (TS=(hydromorphone) OR TS=(dihydromorphinone) OR TS=(palladone) OR TS=(laudacon) OR TS=(dilaudid) OR TS=(hydromorphone hydrochloride)) |
| 2 | (TS=(cancer) OR TS=(tumor-related pain) OR TS=(tumor-associated pain) OR TS=(neoplasm-related pain) OR TS=(neoplasm-associated pain) OR TS=(oncological pain)) |
| 3 | 1 AND 2 |

**Supplemental Table 2. Patient-Controlled Versus Clinician-Controlled Hydromorphone Therapy pooled comparison**

| Outcome | Study | Absolute effect size±SD | | Relative effect size (95CI) | Number of participants |
| --- | --- | --- | --- | --- | --- |
|  |  | Patient-controlled | Clinician controlled |  |  |
| Cancer pain | Bruera et al. 1988 | 29.5±20.4666 | 27.5±17.3098 | Cohen’s d:  -0.202 (-0.621, 0.218) | 236 (2 controlled trials) |
|  | Lin et al. 2020 | 2.7191±0.8042* | 3±0.7965 |  |  |
| Drowsiness | Bruera et al. 1988 | 39±25 | 39±23 | Cohen’s d:  0.102 (-0.143, 0.347) | 234 (2 controlled trials) |
|  | Lin et al. 2020 | 2.35±3.75 | 2±1.50 |  |  |
| Well-being | Bruera et al. 1988 | 42±20 | 46±24 | Cohen’s d: -0.031 (-0.276, 0.214) | 234 (2 controlled trials) |
|  | Lin et al. 2020 | 3.94±2.25 | 3.94±2.25 |  |  |
| Nausea | Bruera et al. 1988 | 22±23 | 26±22 | Cohen’s d: -0.006 (-0.251, 0.239) | 234 (2 controlled trials) |
|  | Lin et al. 2020 | 3±2.25 | 3±1.50 |  |  |
| Appetite | Bruera et al. 1988 | 24±18 | 23±16 | Cohen’s d: 0.010 (-0.235, 0.255) | 234 (2 controlled trials) |
|  | Lin et al. 2020 | 3.94±2.25 | 3.94±2.25 |  |  |

*Significantly lower effect size in the patient-controlled group.
Abbreviations: SD: standard deviation

**Supplemental Table 3. Sustained Release Versus Immediate Release Hydromorphone Therapy pooled comparison**

| Outcome | Study | Absolute effect size±SD | | Relative effect size (95CI) | Number of participants |
| --- | --- | --- | --- | --- | --- |
|  |  | IRH | SRH |  |  |
| Cancer pain | Bruera et al. 1996 | 27±21 | 29±21 | -0.02  (-0.31, 0.28) | 139 (2 trials) |
|  | Hays et al. 1994 | 20±14 | 19±14 |  |  |
| Additional analgesic consumption | Bruera et al. 1996 | 10±8 | 9±7 | 0.03  (-0.27, 0.32) | 139 (2 trials) |
|  | Hays et al. 1994 | 1.0±1.1 | 1.1±1.1 |  |  |

Abbreviations: SD: standard deviation

**Supplemental Figure 1. Sensitivity analysis conducted on studies comparing the reduction of cancer pain between the hydromorphone and morphine groups.**

**
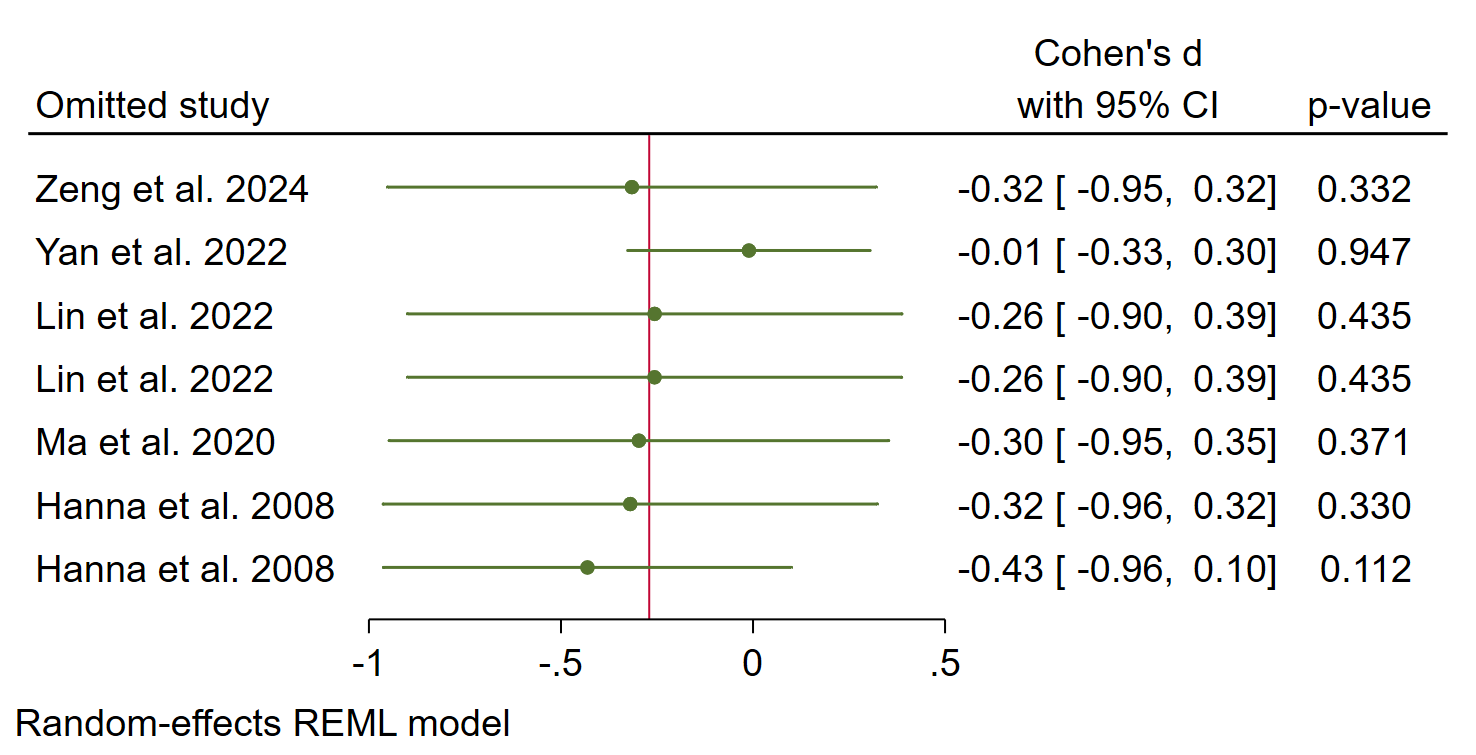
**

**Supplemental Figure 2. The funnel plot of studies comparing the reduction in cancer pain between the hydromorphone and morphine groups**


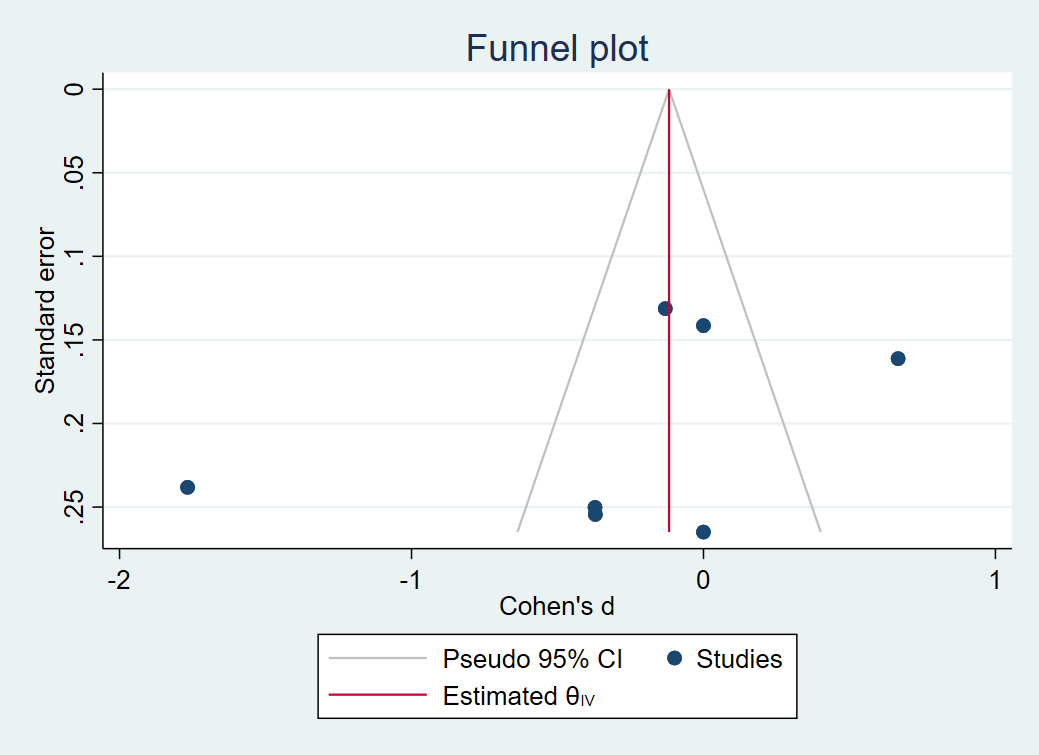


**Supplemental Figure 3. Sensitivity analysis conducted on studies comparing the reduction of cancer pain between the hydromorphone and oxycodone groups.**

**
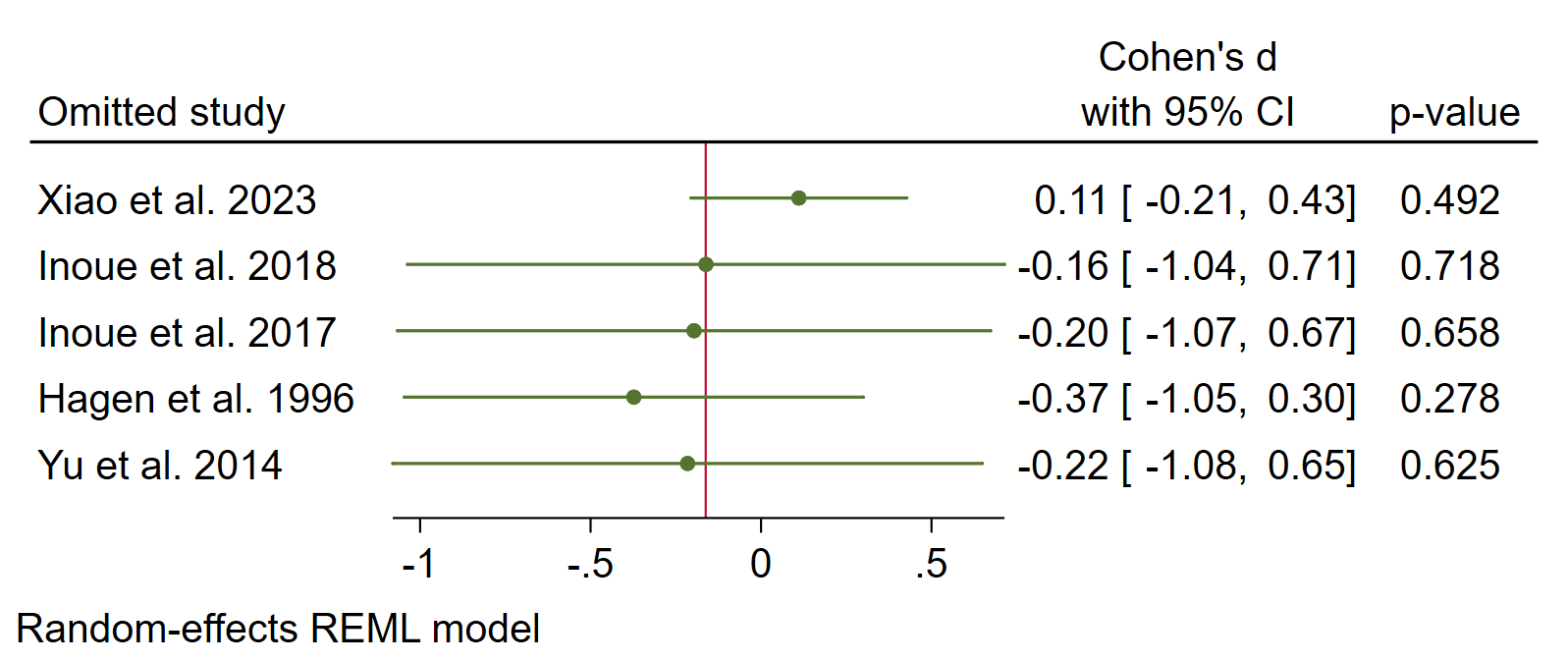
**

**Supplemental Figure 4. The funnel plot of studies comparing the reduction in cancer pain between the hydromorphone and oxycodone groups.**

**
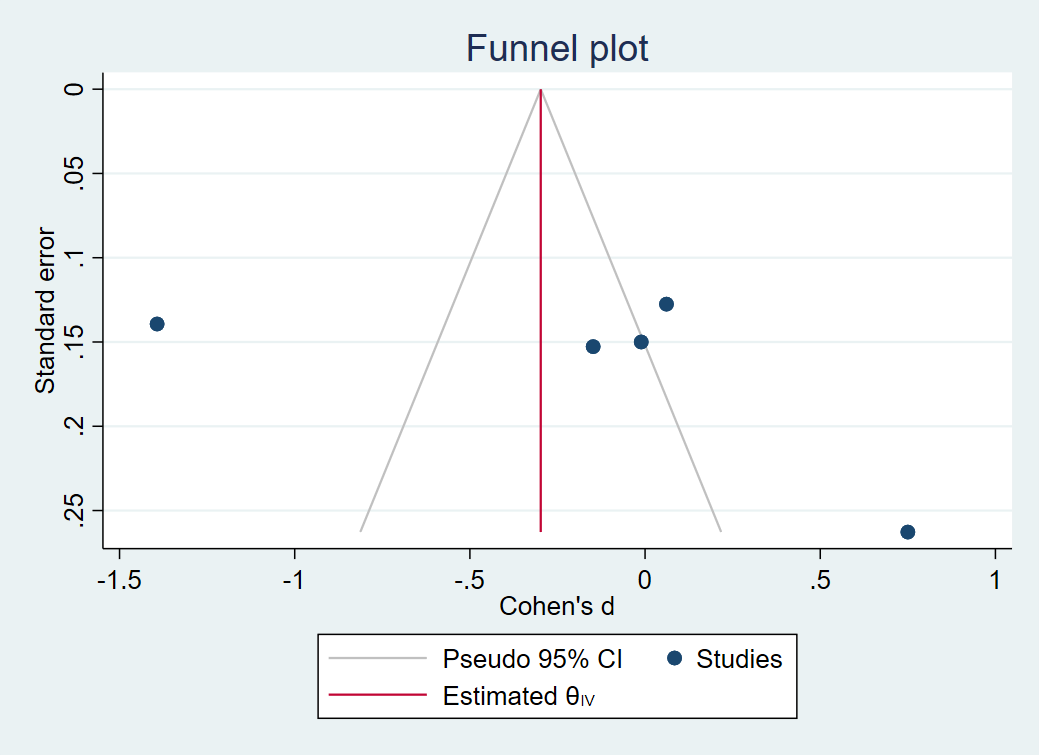
**

**Supplemental Figure 5. Sensitivity analysis conducted on studies comparing the additional analgesic consumption between the hydromorphone and morphine groups.**

**
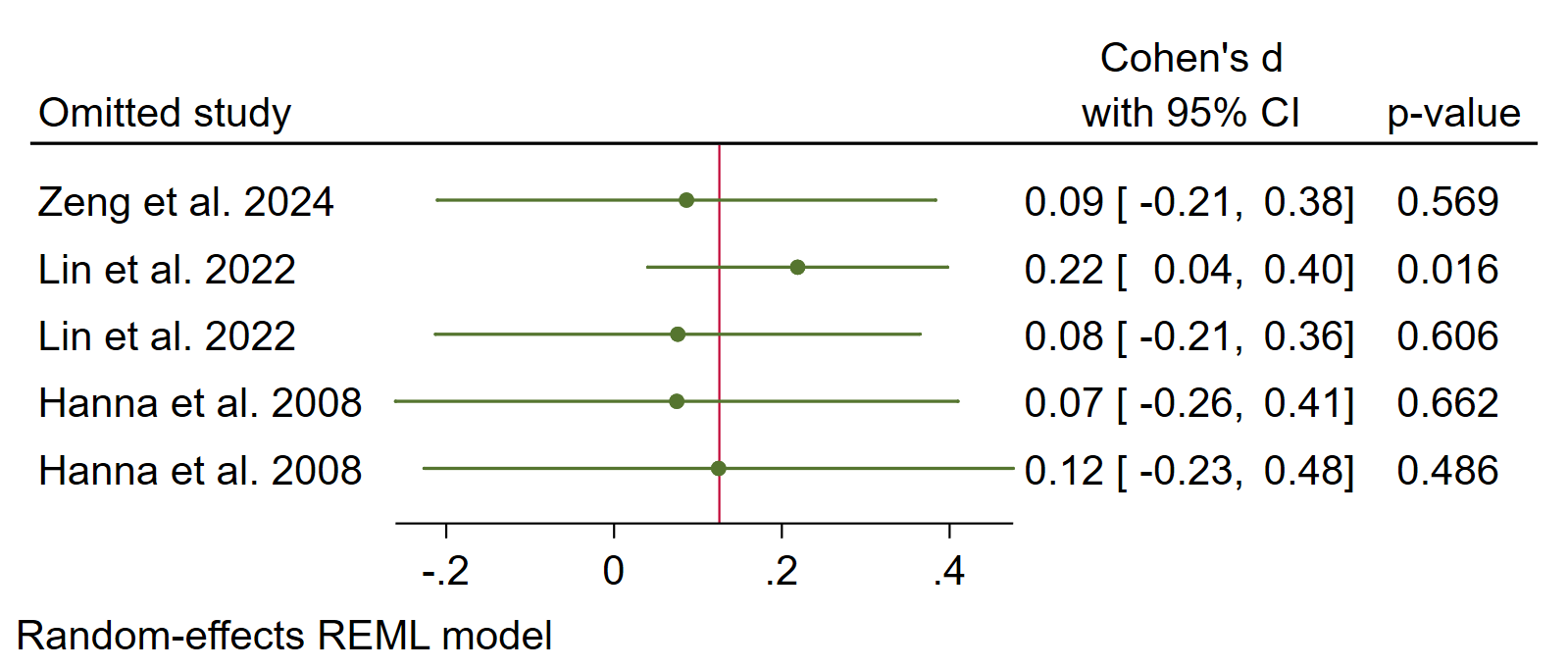
**

**Supplemental Figure 6. Sensitivity analysis conducted on studies comparing the number of breakthrough pain between the hydromorphone and morphine groups.**


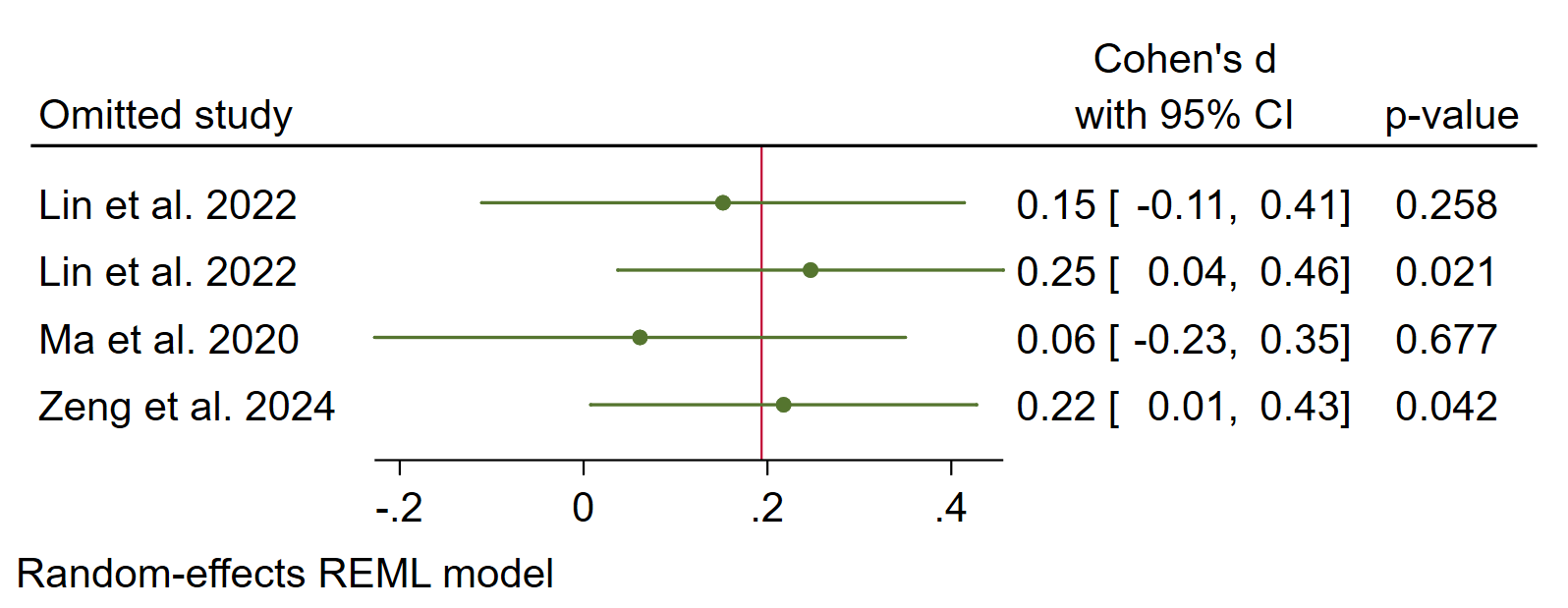


**Supplemental Figure 7. Sensitivity analysis conducted on studies comparing the quality of life between the hydromorphone and morphine groups.**

**
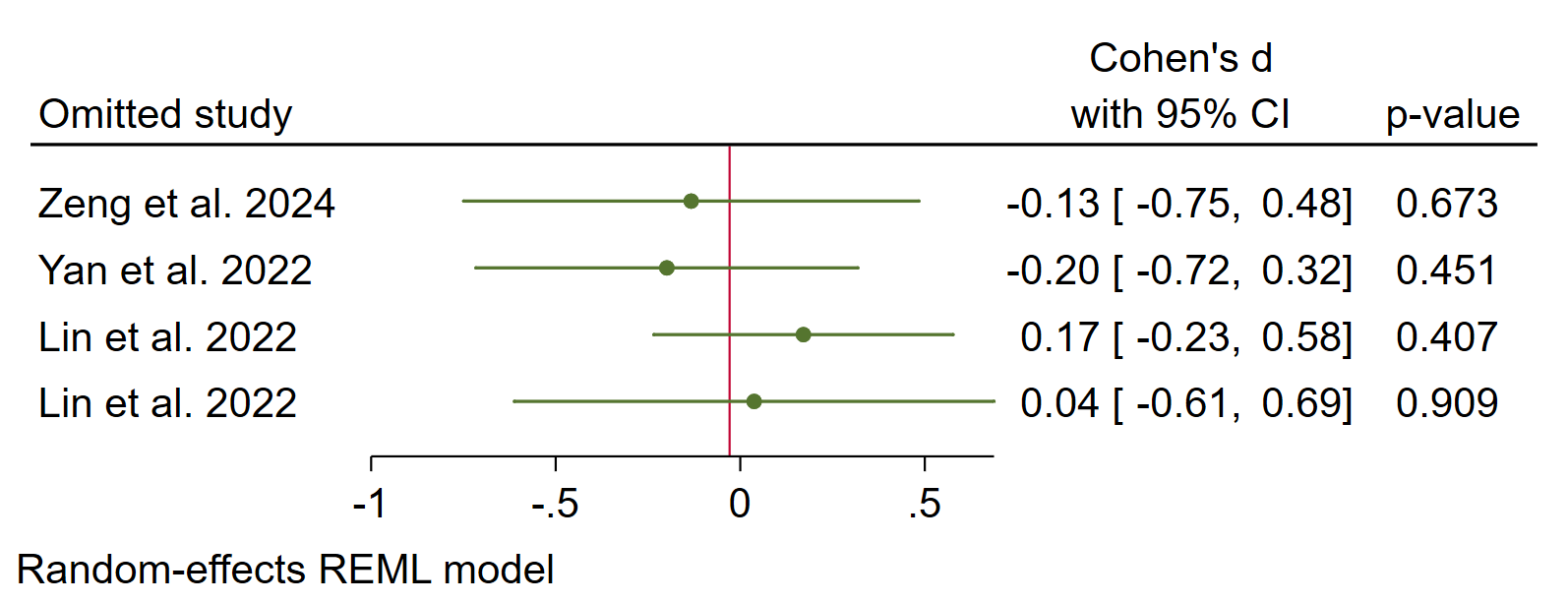
**
